# Percutaneous MR-guided prostate cancer cryoablation technical updates and literature review

**DOI:** 10.1259/bjro.20180043

**Published:** 2019-06-03

**Authors:** Pierre de Marini, Roberto Luigi Cazzato, Julien Garnon, Behnam Shaygi, Guillaume Koch, Pierre Auloge, Thibault Tricard, Hervé Lang, Afshin Gangi

**Affiliations:** 1 Department of Interventional Radiology, University Hospital of Strasbourg, 1 Place de l’Hôpital, Strasbourg Cedex, France; 2 Department of Radiology, King’s College Hospital, Denmark Hill, London, UK; 3 Department of Urology, University Hospital of Strasbourg, 1 Place de l’Hôpital, Strasbourg Cedex, France

## Abstract

Prostate cancer (PCa) is the most common malignant tumor in males. The benefits in terms of overall reduction in specific mortality due to the widespread use of Prostate Specific Antigen (PSA) screening and the advancements in the curative treatments (radical prostatectomy or radiotherapy) appear to have reached a plateau. There remains, however, the questions of overdiagnosis and overtreatment of such patients. Currently, the main challenge in the treatment of patients with clinically organ-confined PCa is to offer an oncologically efficient treatment with as little morbidity as possible.

Amongst the arising novel curative techniques for PCa, cryoablation (CA) is the most established one, which is also included in the NICE and AUA guidelines. CA is commonly performed under ultrasound guidance with the inherent limitations associated with this technique. The recent advancements in MRI have significantly improved the accuracy of detecting and characterizing a clinically significant PCa. This, alongside the development of wide bore interventional MR scanners, has opened the pathway for in bore PCa treatment. Under MRI guidance, PCa CA can be used either as a standard whole gland treatment or as a tumor targeted one. With MR-fluoroscopy, needle guidance capability, multiplanar and real-time visualization of the iceball, MRI eliminates the inherent limitations of ultrasound guidance and can potentially lead to a lower rate of local complications.

The aim of this review article is to provide an overview about PCa CA with a more specific insight on MR guided PCa CA; the limitations, challenges and applications of this novel technique will be discussed.

## Introduction

Prostate cancer (PCa) is a major public health issue. It is the commonest malignant tumor and the second cause of death from cancer in the male population.^1^ The American Cancer Society estimates that in 2018, PCa will be accountable for 29,430 deaths and 164,690 new cases will be diagnosed in the United States alone.^1^

Most cases of PCa are diagnosed in males with no clinical symptoms after a digital rectal examination and a PSA) screening.^2^ This widespread use of PSA screening since the eighties along with the improvements in curative treatments (*i.e.* radical prostatectomy or radiation therapy—RT) has led to a rapid and significant decline in PCa related mortality.^3^ However, these benefits appear to have reached a plateau in recent years especially in the younger male population.^1^ At the same time, the generalization of PSA screening has brought about the issue of overdiagnosis and overtreatment.^4,5^ PCa screening has subsequently led to the treatment of patients with cancers that if left untreated would have potentially not caused any clinically significant consequences during the patient’s lifetime. For this reason, it is essential to consider the adverse effects of the treatment in such patients. Currently, there are strong recommendations for curative treatment in patients with intermediate and high-risk disease.^2,6^ On the other hand, the primary management of patients with low-risk disease and some patients with intermediate risk disease or recurrent disease remain controversial.^7–9^ As a consequence, these patients and those being overdiagnosed and overtreated have highlighted the need for a better definition of clinically significant cancers, and the urge for less invasive and less morbid therapies.^10,11^

Advancements in MRI diagnosis and MR guided minimally invasive treatment for PCa have addressed these issues in part. The development of multiparametric MRI (mpMRI) including several parametric and dynamic sequences (*i.e.* *T_2_* weighted, diffusion-weighted , dynamic contrast-enhanced , and spectroscopy imaging) has allowed precise location of PCa with subsequent possibility for target biopsy and treatment.^12^ On the other side, the availability of miniaturized MR compatible cryoprobes has opened the way to MR guided PCa CA.^13^

The aim of this narrative review is to provide an overview about PCa CA with a more specific insight on MR guided PCa CA; the limitations, challenges and applications of this novel technique will be discussed.

### Why use MRI-guidance for cryoablation?

Among all imaging techniques available in PCa detection, mpMRI has had the most significant impact.^14^ The development of mpMRI has resulted in a substantial improvement in the detection of PCa whilst increasing the confidence in labeling of benign diseases and indolent malignancies. These developments have led to a consensual standardization of MRI acquisition and interpretation methods gathered under the Prostate Imaging Reporting and Data System (PI-RADS) recommendations with its latest version (PIRADS v. 2) release in 2015.^15^ The aim of these recommendations is to improve detection, localization and risk stratification of PCa in order to reduce unnecessary biopsies and treatments. Simultaneously, the improvements in mpMRI have enabled the detection and localization of locally recurrent PCa following previous prostatectomy, RT or focal therapies.^16,17^ In 2014, a consensus panel has established the best practices for the detection of patients eligible for focal therapies.^18^ In spite of advising 3T MRI for PCa detection, the current recommendations leave a place for 1.5T MRI,^15,18^ which is therefore considered appropriate for targeted interventions.

The progress in MR fluoroscopy allows for a precise, multiplanar and real-time needle guidance.^19^ Compared to ultrasound-guided procedures where the needle insertion can only be followed in a single plane, interventional MR-guided procedures allow multiplanar needle guidance. A second and major drawback of ultrasound-guided CA is the poor visualization of the ablated zone due to the angle-shadowing effect of the ice ball.^20^ Since the iceball is solid, it has ultra-short T1 and T2 relaxation times, which results in a significantly high contrast compared to the adjacent soft tissues.^21^ With multiplanar imaging the extension of the iceball can be precisely monitored in order to avoid its contact with non-target organs, such as the rectal wall.^21,22^ Another interventional advantage of MRI compared to ultrasound is the possibility of using alternative pathways to reach the prostate (*e.g.* transperineal or transgluteal) that can be useful in case of unfeasibility of trans-rectal ultrasound (*e.g.* previous ano-rectal resection).^23,24^

There is also a further specific advantage for MRI iceball monitoring in PCa CA. Under ultrasound guidance the operator needs to narrow the recto-prostatic distance in order to optimize image quality, which may subsequently lead to rectal wall injury.^25^ On the contrary, in MR guided interventions, this space can even be widened during the procedure in order to ensure the optimal protection of the rectal wall.^26^

Finally, another potential advantage of MRI over the other imaging techniques is MR-thermometry, which was initially developed for heat-based ablation techniques.^27^ Several recent studies have demonstrated that by using ultra-short echo times, MR-thermometry can be applied also to CA techniques in order to ensure optimal tumor destruction.^28,29^

So far MRI is the only available technique meeting all the required conditions for optimal CA: real time, multiplanar and high resolution imaging for precise tumor location, multiplanar needle guidance and precise iceball monitoring.

### Patient selection for PCa cryoablation

According to the available published studies and the last AUA and NICE guidelines several recommendations on the selection of patients eligible for PCa CA under MRI guidance can be made.^2,6^ Generally speaking this procedure is adapted for patients with a gland-confined intermediate-risk PCa, not eligible to surgery or RT, especially where transrectal ultrasound is not achievable (*e.g.* due to anal pathologies or anoperineal resection). CA can be proposed as a primary treatment in patients unfit or unwilling to undergo standard treatments (*i.e.* surgery and/or RT). Another possibility, which is still not supported by large evidence, is to propose CA as a second-line treatment in patients with recurring gland-confined disease after standard curative treatments (*i.e.* salvage treatment), with the most common case being represented by patients with recurring PCa after RT. Therefore, whole-gland MR-guided CA share the same indications of ultrasound-guided CA: third intention curative therapy for patients with intermediate-risk disease. Moreover, it may be proposed to patients with low-risk disease after having informed the patients about the risks/benefits and the substantial lack of survival benefit when compared with the active surveillance. More specifically, according to the available evidence, whole gland CA seems particularly interesting for patients with recurring disease after RT.^30,31^

Even although the selection of patients eligible for focal therapy remains controversial, the aforementioned considerations along with the evolutions of mpMRI has led to a first consensus for patients’ selection in 2010.^32^ Focal CA should be proposed after having discussed with the patient the risk of recurrence with subsequent need for re-treatment; and the risk of urinary complications, which is probably reduced compared to whole gland CA but far from being negligible.^32^ Patients eligible for focal CA are those with gland-confined intermediate to low-risk disease and with a MR visible biopsy-proven localized PCa.^32^ However, focal CA should be avoided in case of tumor recurrence localized near the urethra because the mandatory use of a urethral warmed catheter may preclude the full ablation of tumor foci.^33,34^

### Whole gland cryoablation

By aiming to destroy all the prostatic tissue, whole gland CA is the closest technique to the standard curative treatments.

Onik et al first described the technique with transrectal ultrasound guidance in 1993.^35^ Since then several improvements have been added to this technique such as urethral catheter warming, fourth generation argon-driven miniaturized (17 gauges) cryoprobes, the use of grid template, biplanar ultrasound probes and thermocouples.^20,36–38^

With the aforementioned technological developments and deployment of several protective measures, the recent studies have demonstrated a significant overall reduction in the side-effects of ultrasound-guided whole gland CA whilst achieving a more efficient freezing of the prostate gland when compared to the earlier publications.^39^ In recent studies about ultrasound-guided CA, the occurrence of urinary/digestive fistula is below 1%.^40^ A large cohort study reported a 12 month complication rate of 9.8% for urinary incontinence, 28.7% for lower urinary tract obstruction, 20.1% for erectile dysfunction and 3.3% for bowel bleeding; and another prospective study reported a 27.7% rate of urinary retention and urgency, 9.4% of catheter related meatal stricture, 9.2% of new-onset erectile dysfunction, 6.1% of urinary tract infection, and 3.8% of hematuria.^40,41^ With the advent of sufficient amount of scientific evidence for long-term oncological efficacy and safety, whole gland CA is now part of the 2014 NICE guidelines and 2017 AUA guidelines as a primary curative treatment.^2,6,40,42,43^ According to these guidelines, the whole gland CA is a third intention curative therapy for patients with good life expectancy (>10 years) and intermediate-risk disease (*i.e.,* PSA 10.0–20.0 ng ml^−1^, Gleason score = 7, cT2b-c) and may be offered also to low-risk patients (PSA <10.0 ng ml^−1^, Gleason Score <7, cT1c-T2a) following discussion of potential complications and absence of survival benefit when compared with active surveillance.^6^

The treatment of recurrent PCa following RT remains controversial with no actual consensus on the best oncologic management to offer to these patients.^44^ A large majority of them will be treated by androgen deprivation therapy (ADT) with its procession of side-effects.^45,46^ Several teams have already included CA in their salvage therapeutic armamentarium.^47^ As a salvage treatment, CA can be an effective oncological tool, which may allow ADT postponing.^48^ Thus, the whole gland CA is a validated curative primary treatment as well as an emerging technique in the salvage setting.

In bore percutaneous whole gland CA was first described by Gangi et al. in 2011.^30^ This study included 11 patients (8 primary treatments and 3 post-RT recurrent cases) who were considered to have contraindications for standard therapies or who rejected them. The procedures were undertaken in a large, closed-bore, 1.5T MR scanner. The operator considered full technical success in 10/11 cases. In the last case, the anterior part of the gland remained unfrozen. The mean follow-up period was 15 months (range: 1–25 months). All patients showed PSA reduction with a mean PSA nadir of 0.33 ng ml^−1^ (range: 0.02–0.94 ng ml^−1^). Six minor and one major complications were reported. The major complication was a rectourethral fistula in one of the first patients, which spontaneously healed by the 3 month follow-up. This same patient was also the sole case of residual or recurrent disease in the study follow up. The minor complications included one case of urinary tract infection, three cases of transient dysuria and urinary retention, one case of hematuria and one case of scrotal pain. The results of this study have been recently expanded by De Marini et al who investigated the safety and the oncologic results at mid-/long-term in a population of 30 consecutive patients (18 primary treatments; 12 salvage treatments) including the first 11 patients previously reported.^49^ In this larger experience, the overall local progression-free survival (LPFS) in the whole population was 92.0%, 75.7% and 69.4% at 1-, 3- and 5 year follow-up, respectively. When stratified by treatment type, 1-, 3- and 5 year LPFS was 88.9%, 75.2%, and 67.7% in the primary treatment group and 100%, 75.0% and 75.0% in the salvage treatment group, respectively. The results were surprisingly better in the salvage therapy group although the difference was not significant. The complication rate remained significant (60%) with six reported major complications (20%); among them, five patients required surgical/interventional management, including three TURP for persistent obstruction/retention, one endoscopic urethrotomy for urethral stricture and one artificial sphincter surgery for incontinence. The remaining major complication corresponded to the urethra-rectal fistula previously described by Gangi et al. Although the complication rate was high, it is worth to note that this study included patients treated over a 10 year period during which the technique was continuously adapted; in fact, the complication rate was substantially reduced between the first 15 patients (72 %) and the last 15 patients (47 %) .

Kinsman et al also conducted a similar intervention on four patients with contraindications to standard therapies including one with recurrent PCa after RT.^31^ All four patients in the series had undetectable PSA at 3–6 months post-CA and PSA remained undetectable at 12 months for all of the cases. 5 years follow-up data were available in two patients with PSA remaining undetectable. For the last two patients of this series one had an undetectable 3 years post-ablation PSA and the last one passed away 3 years post CA due to unrelated acute liver failure. In this study, patients were questioned about urinary symptoms before and after CA. Two patients reported no change in urinary symptoms or erectile function. One patient reported a new pad-free incontinence and one other had increased erectile dysfunction.

**Figure 1.**
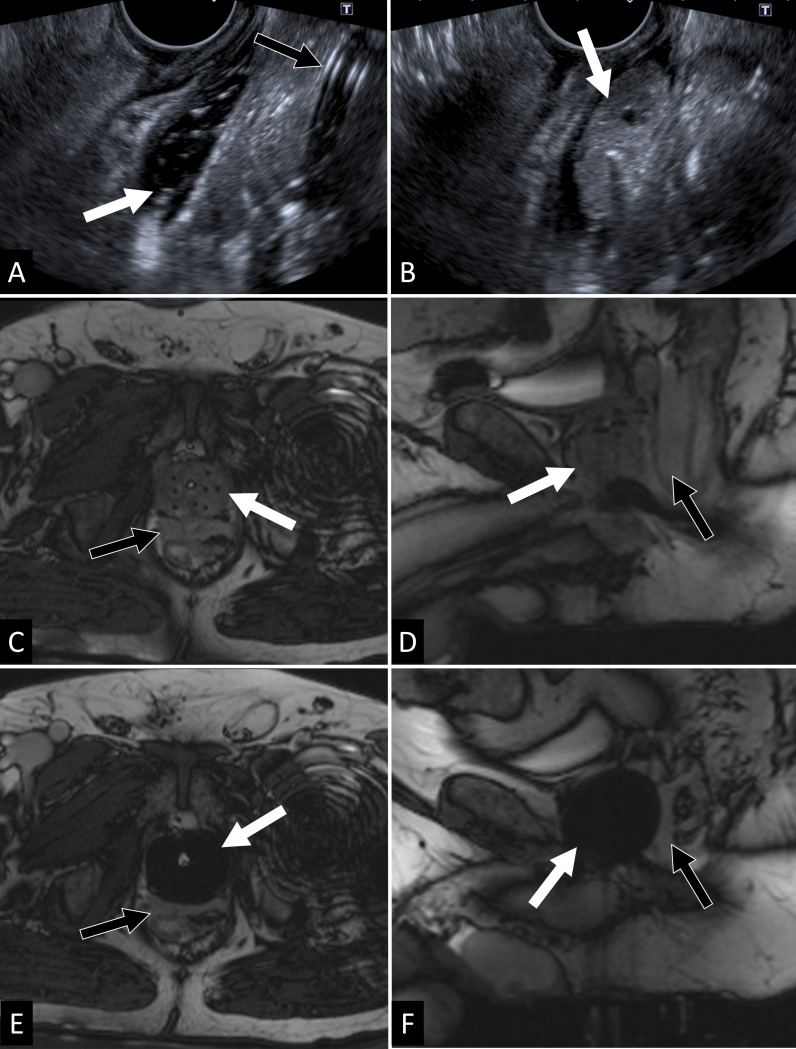
MR-guided whole gland CA as a primary treatment in a patient with a multifocal Gleason 4 + 3 PCa. A transperineal hemodissection of the Dennonvillier’s fascia is firstly performed under transrectal ultrasound guidance (A, B). The midline is repaired thanks to visualization of the urinary probe (black arrow in A). First some saline is injected to confirm the good positioning of the needle (white arrow showing the anechoic area in A). Afterwards autologous blood injection allows formation of a clot (arrow in B). Axial and sagittal *T*_2_ TRUFISP acquisitions showing final cryoprobe positioning (arrows in C and D) and maximal Iceball extension (arrows in E and F). The clot in the recto-prostatic space is well visible in *T*_2_ with an hypersignal (black arrows in C, D, E and F). MRI allows here to ensure complete gland coverage with sufficient margins while controlling the distance between the ice ball and the rectal anterior wall (arrow head). CA, cryoablation; PCa, Prostate cancer.

Currently, at our institution (University Hospital of Strasbourg, France) the procedure is performed under general anesthesia on a 1.5T in-bore MRI-unit. Following ultrasound-guided hemodissection of the Denonvilliers' fascia to reduce the risk for CA-mediated injury of the anterior rectal wall, the patient is transferred to the MRI room.^26^ After a first multiplanar *T*_2_WI assessment of the gland, several cryoprobes are inserted trans-perineally through a rigid grid placed on a dedicated device (Uni-Lift Prostate Intervention Device,NORAS GmbH, Höchberg, Deutschland). The probes are spaced away 1 cm one from the other and are 5 mm within the capsule. The iceball is then monitored by iterative acquisition of *T*_2_-weighted multiplanar images (Figure 1).

### Focal cryoablation

The advances in MRI allowing precise localization of the PCa has led to the idea of focal targeted therapy. This option is particularly counterintuitive, as PCa is frequently multifocal.^50^ The natural history of this slow evolving cancer suggests that though multifocal, the prognosis of the disease is mainly dictated by the largest lesion, which most often is also the highest-grade lesion.^51,52^ The theoretical principle underlying the focal therapy is that the treatment of this index dominant lesion may significantly alter the natural history of the disease. The long-term relevance of the secondary tumors and their potential of extracapsular spread however, remain somewhat uncertain. The resultant hypothetical benefit of this tissue sparing strategy is to limit the collateral damage to urinary, bowel and erectile functions associated with whole gland PCa therapies. The ultimate idea of this strategy is to offer a treatment that minimizes the impact on the quality of life while improving the oncological prognosis compared to active surveillance or standard therapies thus, potentially opening the way for treatment also in low-risk patients.^33^ Moreover, these considerations can also be extended to the salvage treatment.^53^

Until now, only a few studies about focal CA as a primary treatment have been published.^54–56^ In these studies, focal CA corresponds more to an ltrasound-guided hemi-gland ablation confined to the lobe where the disease is predominant according to mpMRI rather than a truly targeted treatment; and to the best of our knowledge, there are no published papers about in bore MR-guided focal CA as primary PCa treatment. Valerio et al recently published a series including 23 patients undergoing MRI-transrectal ultrasound fusion focal CA.^57^ The MRI-ultrasound fusion was deemed inadequate in two patients thus, demonstrating the limits of this approach in terms of procedural guidance. The MRI–ultrasound fusion was efficient in 18 patients, amongst which focal CA was the primary treatment for 14 patients. In this series, Valerio et al reported 10 adverse events and demonstrated no residual cancer in the ablation zone on late MRI (between 6 and 12 months) in all 18 patients. However two patients showed disease progression on the contralateral lobe. This targeted MR imaging–ultrasound fusion approach is undoubtedly even more challenging to achieve in salvage treatments given the small and anatomically altered prostate. In this scenario (in-bore) MR guidance may prove advantageous.

In 2013, Woodrum et al reported their initial experience on focal CA for local recurrence after radical prostatectomy.^58^ This study included 18 patients placed into two equal groups. Nine patients were treated with cryoprobes placed 1 cm apart with two freeze-thaw cycles (Group 1: standard treatment group). The other nine patients received cryoprobes placed 0.5 cm apart with three freeze-thaw cycles and a variation of the urethral warmer temperature according to the location of the lesion (Group 2: aggressive treatment group). All patients were treated inside a wide bore 1.5T MRI. The authors demonstrated an immediate and significant PSA reduction in both groups, but a longer lasting PSA reduction was noted in the aggressive treatment group. During the follow-up period no patient in Group 2 showed signs of local recurrence whereas in Group 1, four patients had local recurrence in the prostate gland and two had recurrence outside the prostate bed. Conversely the authors reported higher complications in the aggressive treatment group with one patient requiring an artificial urethral valve. Although this study is very informative on the potential strategy to adopt, it is limited by its short follow-up time and the number of patients lost to follow-up (PSA data at 12–15 months were only available for five patients in Group 1 and 4 patients in Group 2).

The same year, a similar pilot study was conducted by Bomers et al, who prospectively included a10 patients with a history of pelvic RT : 9 with PCa recurrence after RT and 1 patient with newly diagnosed PCa but a history of testicular cancer.^59^ In order to protect the adjacent tissue, a urethral and a rectal warmer were systematically used. The procedure was technically feasible in all cases. Only one case of early complication (urinary retention) was reported. Eight patients were discharged the following day after the procedure and the two others were admitted in the hospital for only 3 days. These data need to be put in perspective with the usual 8–14 days of hospital stay following a salvage radical prostatectomy and the need for several admissions for salvage RT.^60^ In their study, Bomers et al reported two cases of local recurrence at 6 months, both of whom were treated by repeated MRI guided CA. One other patient had a pelvic lymph node recurrence treated by ADT. During the follow-up, three patients reported hematospermia, hematuria and scrotal pain and two patients had urinary retention requiring self-catheterization, one of whom required surgery for a urethral stricture.

More recently Overduin et al conducted a retrospective analysis of data on 47 patients treated by percutaneous MR-guided focal CA of biopsy-proven local recurrence of PCa after primary RT.^61^ The main focus of this study was on the need to obtain sufficient iceball margins. In their follow-up period, the authors reported 23 cases of local tumor progression after a median follow up of 12 months (range 3–42). They also demonstrated that significantly better local tumor control could be achieved in the patients also receiving ADT and when the iceball margins are at least 5 mm beyond the border of an MR-visible recurrent prostate tumor. The authors stressed the difficulty in obtaining sufficient iceball margins for the tumor situated at the posterior aspect of the prostate due to the close proximity of the rectal wall and those with a volume in excess of 1 ml. This difficulty may be explained by the use of a rectal balloon as thermoprotective measure, which tends to reduce the distance between the prostate and the rectal wall.

**Figure 2.**
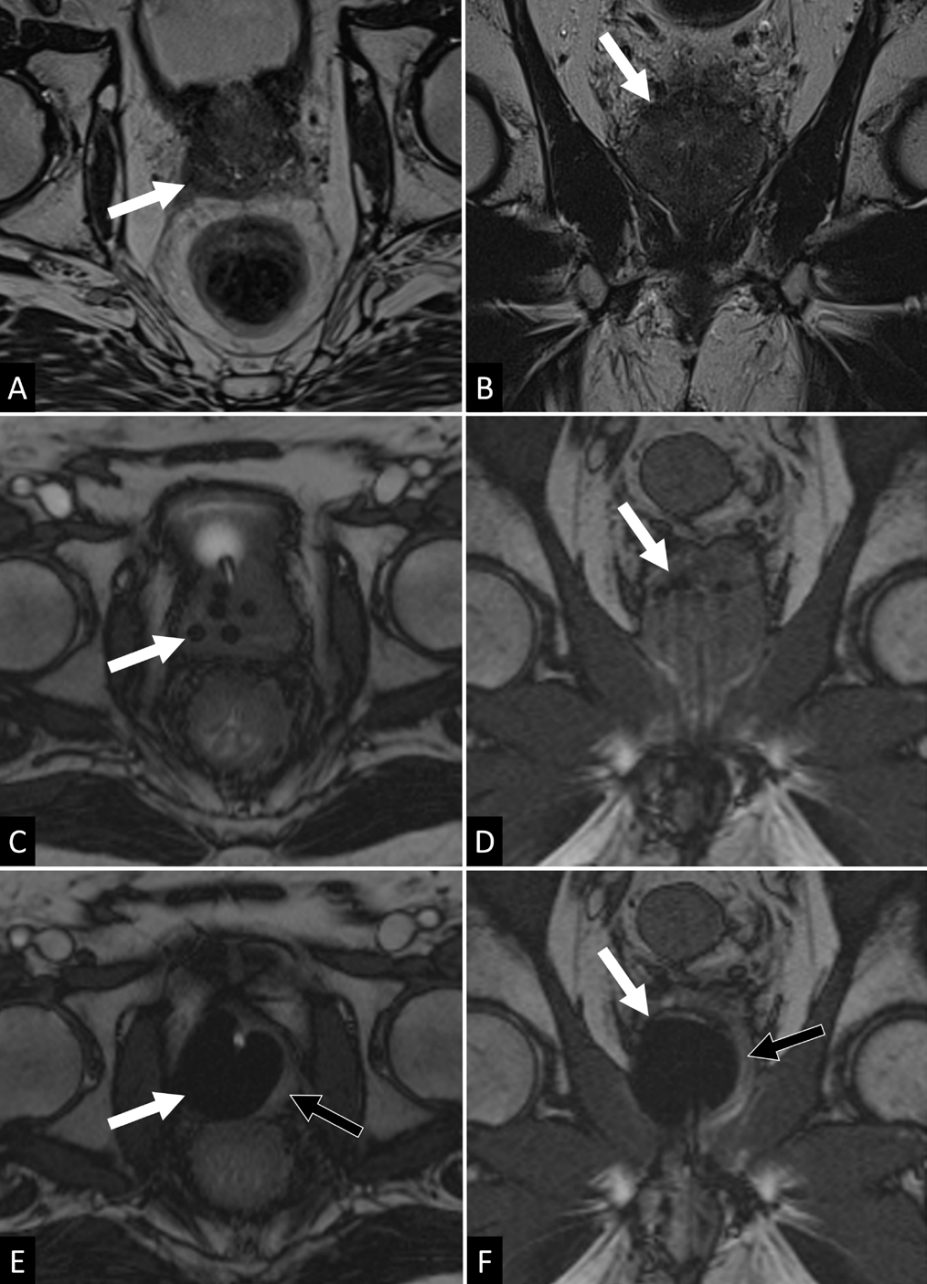
Right lobe CA of the prostate in a patient with a histology proven (Gleason 4 + 3) post-RT focal recurrence. The pre-procedural axial and sagittal *T*_2_ SPACE acquisitions (A,B) allow the identification of the tumor recurrence as a discrete peripheral *T*_2_ hypointense area of the right prostate base (arrows). Repeated *T*_2_ TRUFISP sequences are then used for needle insertion and positioning and also for iceball monitoring during the procedure. Axial and coronal sequences illustrate here final needle positioning (arrows in C and D) and maximal iceball extension (arrows in E and F). In this procedure the iceball covers the entire right lobe while sparing part of the left lobe (black arrows in E and F) and thehomolateral neurovascular bundle. RT, radiation therapy.

Figure 2 shows an example of focal treatment (*i.e.,* right hemi-ablation) performed at our institution.

### Protective measures needed during PCa CA

During MRI-guided CA the ice ball apparent to the operator can be larger than the oncologically effective ablation zone. In fact, on the iceball margins, clearly distinguished on MRI, a temperature of 0°C is often recorded. On the other hand, studies have demonstrated that the lethal freezing temperature for cancer tissue is around −40°C.^62^ Therefore, the lethal freezing margins may actually be situated approximately 1 cm within the iceball edge.^63,64^ Obtaining sufficient margins is particularly difficult to achieve within the small space of the male pelvis where the proximity of the iceball to the rectum, the external urethral sphincter and the neurovascular bundle must be particularly monitored and spared. These limitations however, are not insurmountable. A way to optimize the interprostatorectal space has been recently described by Garnon et al. (Figure 1) and it may also be feasible to perform dissection of the prostate apex from the external urethral sphincter.^26^

In PCa CA the use of a heated urethral catheter is necessary in order to prevent damage to the external urinary sphincter, which would otherwise have catastrophic consequences on the patient's quality of life. This can impose a limiting factor in achieving a total ablation of the PCa. In a small study Pisters et al found that 2 out of 7 patients had remaining viable periurethral cancer.^65^ More recently Favazza et al investigated the impact of the urethral warmer on the adjacent tissue.^34^ In their study, they outlined that the urethral warmer is mandatory in order to prevent lethal damage to the urethral surface. Moreover, in order to effectively perform CA, the cryoprobes should not be more than 1 cm apart.

Several authors have also suggested the use of MR thermometry to obtain the exact thermal isotherms inside the iceball.^28,29^ However, although the acquisition time of these ultra-short TE sequences is short (about 1 min), the need for calibration curves and pre-treatment image processing limit their use on a large scale in routine clinical practice. To address these drawbacks, referenceless proton resonance frequency shift thermometry has been developed by some teams but it has only been evaluated on the heat generating ablative techniques such as high intensity focused ultrasound.^66,67^ Similarly Overduin et al suggested the use of short echo time *T*_1_ sequences to monitor the cooled but unfrozen area.^68^ This method seems to be simpler and easier to access but only investigates the iceball risk area but not necessarily the oncologically effective area.

Another way to control the safe margin of the iceball is the use of MR compatible thermocouples. These thermocouples can be placed near the organs to be protected such as the anterior rectal wall, the external urinary sphincter and the neurovascular bundles.^69^

### Limits and perspectives

To date, robust scientific evidence supports the use of whole gland or focal CA in the treatment of PCa.^6,42^ Focal CA is increasingly used due to potentially lower complication rates, preserved sexual function and satisfactory local control when compared to the whole-gland CA.^33^ Very few studies comparing focal to whole gland CA are available. In 2012, Ward et al analyzed the Cryo On-Line Data (COLD) registry (which included ultrasound-guided focal CA where focal CA could vary from real focal CA to hemi-gland ablation) and noted that the safety profile was slightly superior in focal than whole gland CA but the preservation of the sexual function was not as superior as expected.^56^ In 2017 Tay et al compared the results between 166 pairs of males with intermediate risk disease treated by ultrasound-guided focal or whole-gland CA and a significantly better sexual function at 12 months could be demonstrated for focal CA.^70^ The limited follow-up period of this study (12 months) however, limits the conclusion on the safety profile of CA. In salvage treatments, the safety benefit of focal CA is even less evident.^53^ However Valerio et al reported that by using MRI-transrectal ultrasound fusion, focal CA caused no deterioration of the erectile function or lower urinary tract symptoms from the baseline.^57^ In the only study comparing ultrasound-guided salvage focal CA to ultrasound-guided salvage whole-gland CA, whilst reporting a trend towards less complications for focal CA, this did not achieve statistical significance.^71^ Finally, a recent prospective study on the safety and oncological efficacy of both ultrasound-guided focal and whole gland CA, whilst not aiming to compare the safety profile of focal and total CA, suggested that the difference in safety profile might be inferior than expected.^41^

The focal approach seems to offer a comparable local tumor control when compared with the whole-gland ablation in patients with low and intermediate risk-disease in ultrasound-guided series.^56,70–72^ However some authors may propose a second focal CA to their patients in order to achieve this goal.^59,61^ As CA is not a radical treatment, the definition of recurrence following CA is particularly treacherous and can result in heterogeneous definition of recurrence across studies.^41,73^ This heterogeneity renders conclusions on oncological efficacy problematic. By mimicry with RT, the most consensual definition of local recurrence following the whole-gland CA seems to be outlined by the Phoenix criteria. These criteria, however, are not applicable to focal therapies.^74^ Thus, it is likely that because of these totally different theoretical approaches, the superiority of the whole-gland approach over the focal approach (or vice versa) can only be assessed by the studies that carry out comparative monitoring over a long period.^75^

Though higher than expected (Table 1) the complication rate of in-bore MRI interventions remains acceptable if we consider that many of the patients included in these studies were not eligible for standard therapies and presented with several comorbidities. In our current experience, (in-bore) MR-guided CA is proposed as a primary or salvage treatment to patients with good life expectancy not eligible for or unwilling to undergo standard treatments. Our current institutional (University Hospital of Strasbourg, France) preference is for whole-gland CA, in line with the current NICE and AUA guidelines. All patients benefit from two urethral sphincter protection methods (*i.e.* urethral warmer and urethral sphincter thermometry) along with hemodissection of the Denonvilliers' fascia to protect the anterior rectal wall thus, allowing, at the same time, optimization local tumor control at this level without an increased risk of rectal wall injury.

**Table 1.** Summary of the main available studies reporting about in-bore MR-guided PCa CA

Study	Year	Whole gland/ Focal	Gleason Score range	Mean Age	Number of patients	Primary / Salvage treatment	Follow-up duration	Recurrence rate	Overall complication rate
De Marini et al.^49^(including Gangi et al.^30^	2019 (2012)	Whole gland	5–8	73	30	18/12	3.8 years [2 days-8 years]	7 [23.3%]	18 [60%]
Kinsmann et al.^31^	2017	Whole Gland	6–7	64	4	3/1	12 months to 5 years	0 [0%]	2 [50%]
Woodrum et al.^58^	2013	Focal	5–9	67	18	0/18	12–15 months	6 [33%]	NA
Bomers et al.^59^	2013	Focal	6–8	67	10	1/9	12 months	3 [33%]	5 [50%]
Overduin et al.^61^	2017	Focal	NA	66	47	0/47	12 months^3–42^	23 [49%]	NA

NA, not available.

## Conclusion

Amongst the emerging therapies within the therapeutic arsenal of PCa, CA is probably the most established technique. The debate between opting for the whole gland versus focal CA remains open. Focal therapies provide an attractive option, apparently limiting the complications rate; nevertheless, focal CA remains a non-radical treatment given the propensity of PCa to be multifocal. On the other hand, whole gland CA is the technique that is the closest to the gold-standard curative treatments such as radical prostatectomy, and is currently included into international guidelines. Both these options are proposed under imaging guidance and traditionally ultrasound guidance has been used. However, several authors have published reports on MRI-guided CA highlighting all its major strengths, especially in terms of monitoring capabilities. Therefore, it is likely that in the near future MRI-guided PCa will play a major role in the treatment of patients with intermediate (or low-) risk disease unsuitable for standard treatments or needing salvage treatment. Further robust prospective studies are needed to clearly establish the right role of whole gland and focal CA of PCa unde MRI-guidance.
